# Integrate single-cell and transcriptome analyses to explore the prognostic genes related to TRPM4 in bladder cancer

**DOI:** 10.3389/fbioe.2026.1773551

**Published:** 2026-04-13

**Authors:** Qi Zhao, Zitong Qin, Runzhang Liu, Kangwei Zuo, Chenghao Guo, Suoshi Jing, Weiping Li

**Affiliations:** 1 The First School Of Clinical Medicine, Lanzhou University, Lanzhou, China; 2 Department of Urology, The First Hospital of Lanzhou University, Lanzhou, China

**Keywords:** bladder cancer, prognostic genes, risk model, single-cell analysis, TRPM4

## Abstract

Bladder cancer (BLCA) is a common malignancy of the urinary system, yet the therapeutic relevance of transient receptor potential cation channel subfamily M member 4 (TRPM4) remains unclear. By integrating single-cell and whole-genome transcriptomic data, this study revealed significant transient receptor potential cation channel subfamily M member 4 (TRPM4) overexpression in bladder cancer (BLCA) (*p* < 0.05), particularly in epithelial cells. Intersection analysis identified 220 candidate genes (7,808 DEGs1, 4,683 DEGs2, and 4,802 key cell module genes). A risk model was constructed comprising six screened prognostic marker genes, namely, protein unc-93 homolog B1 (UNC93B1), family with sequence similarity 193 member B (FAM193B), protein O-glucosyltransferase (POGLUT3), fibrillin-1 (FBN1), microtubule-associated protein 1B (MAP1B), and RUNX family transcription factor 2 (RUNX2). The model demonstrated marked differences among the risk groups. Gene set enrichment analysis revealed significant disparities in key pathways, including the melanoma pathway (*p* < 0.05). Furthermore, immune infiltration analysis has identified 12 distinct immune cell types, including naive B cells, which showed a *p* < 0.05 distribution. The observed distribution was uneven. In the drug sensitivity analysis, 112 drugs (including WZ3105; *p* < 0.05) showed differential responses, and UNC93B1 showed high positive expression in BLCA tissues (positive cell proportion > 75%). Our studies confirmed that TRPM4 has significant prognostic value and is a potential novel diagnostic and therapeutic target for BLCA.

## 1 Introduction

Bladder cancer (BLCA), a major category of urinary system malignancies, is a significant global health issue. Statistical analyses indicate that over 570,000 new cases of BLCA are diagnosed annually worldwide, resulting in approximately 210,000 deaths per year and posing a severe threat to public health (Yang et al., 2024). Recent research into the molecular mechanisms of BLCA has expanded beyond traditional protein-coding genes to include non-coding RNA regulation, epigenetic remodeling, and interactions within the tumor microenvironment. Studies have shown that long epithelial alu-interacting differentiation-related (LEADR) non-coding RNA is subject to epigenetic regulation by p63, with a notable decrease in expression observed in muscle-invasive cells (Barnaba et al., 2025). The nuclear expression of LEADR enhances sensitivity to interferon signaling, thereby revealing novel antitumor immune mechanisms. In addition, exosome-derived long non-coding RNA (lnc-TAF12-2:1) has been demonstrated to promote tumor progression via the miRNA-7847-3p/ASB12 axis (Chen et al., 2025). Single-cell technologies represent a significant advancement in this field. Single-cell chromatin accessibility mapping delineates the epigenetic regulatory landscape of BLCA (Xiao et al., 2025). Spatial transcriptomics of lymph node metastases identified the myoCAF_PLXDC1 fibroblast subpopulation associated with poor prognosis and its colocalization with integrin subunit alpha 8-positive (ITGA8+) endothelial cells (Wang et al., 2025a). Further, multiple endocrine neoplasia type 1 (MEN1) has been shown to promote cancer cell proliferation by activating the wingless-related integration site (Wnt)/β-catenin pathway, with the inhibitor BAY-155 demonstrating anticancer effects both *in vitro* and *in vivo* (Shi et al., 2025). In addition, endoplasmic reticulum oxidoreductase 1 alpha (ERO1A) may influence tumor cell invasiveness via the arachidonate 5-lipoxygenase (ALOX5)/janus kinase (JAK)/signal transducer and activator of transcription (STAT)/autophagy signaling axis (Huang et al., 2025). The dynamics of single-cell communication in T-cell immunotherapy further underscore the pivotal role of immune microenvironment remodeling (Liu et al., 2026b). These advances indicate that BLCA evolution results from the coordinated action of multilevel, multidimensional regulatory networks. Consequently, approaches leveraging recent advancements in BLCA research, integrating multiomics data, thoroughly examining core molecules linked to tumorigenesis, metastasis, and immune evasion, and constructing robust prognostic prediction models are of considerable scientific and clinical significance for enhancing the translation of precision medicine in BLCA.

As a non-selective cation channel, TRPM4 is pivotal in numerous physiological processes as it modulates intracellular calcium signaling and stabilizes membrane potential (Autzen et al., 2018). Recent studies have identified a strong correlation between aberrant TRPM4 expression or dysfunction and the progression of various malignant tumors, including breast, colorectal, and liver cancer (Dai et al., 2025). TRPM4 is pivotal in modulating multiple malignant tumor cell phenotypes, including proliferation, apoptotic resistance, invasion, and metastatic dissemination (Wang et al., 2022a). BLCA studies have demonstrated that TRPM4 expression is notably higher in BLCA cells versus normal bladder tissue in mice (Dienes et al., 2021). Further, high TRPM4 expression is closely associated with tumor stage, lymph node metastasis, and poor prognosis in patients (Dienes et al., 2021). However, the specific molecular mechanisms involving TRPM4 in BLCA metastasis, including how it may influence downstream signaling pathways and influences the tumor microenvironment, remain to be elucidated. Consequently, conducting extensive research on the function and mechanisms of TRPM4 in BLCA metastasis may offer novel theoretical foundations for developing targeted therapeutic strategies and improving the prognosis of patients with BLCA.

Single-cell sequencing technology, also known as single-cell RNA sequencing, is unparalleled in the field of transcriptomics (Jung et al., 2023). This approach facilitates comprehensive, unbiased cell studies, allowing researchers to characterize complex biological processes at the cellular level. Single-cell RNA sequencing provides unprecedented insights into the diverse cell types and functional profiles within complex organisms by providing data on transcriptional heterogeneity and complexity at single-cell resolution (Jovic et al., 2022). This technology enables the precise capture of subtle disparities among cells, thereby offering unique insights into cell differentiation, development, and disease mechanisms (Jovic et al., 2022).

The present study identified differentially expressed genes (DEGs) in BLCA using transcriptomic data from public databases, as well as DEGs in high- and low-TRPM4 expression groups. Further, we identified critical cells in BLCA using single-cell sequencing data. The application of high-dimensional weighted gene coexpression network analysis (hdWGCNA) facilitated the identification of module genes in key cells. Our findings indicate that TRPM4 is a promising prognostic marker and therapeutic target in BLCA. In addition, quantitative real-time polymerase chain reaction (qRT–PCR) revealed the expression of prognostic genes in BLCA tissues, laying the foundation for further investigations into the pathogenesis of BLCA and enhancing clinical prognostic assessment systems.

## 2 Materials and methods

### 2.1 Data collection

BLCA transcriptomic and corresponding clinical data were procured from The Cancer Genome Atlas (TCGA)-BLCA project via the UCSC Xena database (http://xena.ucsc.edu/). The dataset comprised 19 normal adjacent tissues (NATs) and 409 tumor samples, of which 404 had complete survival records, collectively forming the training cohort for this study (accessed 10 April 2025).

To validate the findings, we obtained the GSE13507 dataset (Platform: GPL6102) from the Gene Expression Omnibus (GEO) database (https://www.ncbi.nlm.nih.gov/geo/), providing a validation set of 165 BLCA samples with complete survival and expression data. For the single-cell analysis, we obtained the scRNA-seq dataset GSE222315 (Platform: GPL24676), containing 9 BLCA tumor and 4 NAT samples.

### 2.2 Analysis of the role of TRPM4 in BLCA

To discern DEGs in the TCGA-BLCA cohort, we compared the tumor group against the normal group. The analysis was conducted with the “Deseq2” package (v1.4.2) (Love et al., 2014), applying the thresholds of |log2FC| > 0.5 and *p* adj <0.05 (corrected with the Benjamini–Hochberg (BH) method) for screening. The volcano plot and heatmap for DEGs1 were generated using the “ggvolcano” (v0.0.2) and “ComplexHeatmap” (v2.14.0) packages, respectively (Gu et al., 2016). Both visualizations were annotated with the top 10 significantly upregulated and downregulated genes, ranked by |log2FC|. Subsequently, to visualize the top 10 upregulated and downregulated DEGs1, boxplots were generated with the “ggplot2” package (v3.4.1) (Wang et al., 2022c). TRPM4 gene expression data were retrieved from the normalized TCGA-BLCA dataset. The Wilcoxon test was applied to identify DEGs between the tumor and normal tissues (*p* < 0.05). Concurrently, patients were stratified into high- and low-TRPM4 expression groups according to the median value to clarify intergroup differences. The Wilcoxon test and the “DESeq2” package (v1.4.2) were employed to analyze gene expression differences and identify TRPM4-associated DEGs2 (i.e., high-vs. low-expression group), respectively, both with a significance threshold of *p* < 0.05, with the latter additionally requiring |log2FC| > 0.5. To visualize DEGs2, we generated a volcano plot (“ggvolcano” v0.0.2) and a heatmap (“ComplexHeatmap” v2.14.0), labeling the top 10 significant genes. Boxplots were created to visualize their expression (“ggplot2” v3.4.1). To assess TRPM4’s prognostic role, we compared patient groups (“survminer” v0.4.9; log-rank test, *p* < 0.05) using survival time and status. The association was visually represented using Kaplan–Meier (K–M) survival curves.

### 2.3 Methods for single-cell data processing

In the GSE222315 dataset, batch effects were processed using the RunHarmony function from the Harmony package (v1.2.1), merging NAT and BCa samples into a single complete Seurat pair. To perform quality control on the single-cell dataset GSE222315, we applied the “Seurat” package (v5.1.0) with the following criteria: nFeature_RNA between 300–5,000, mitochondrial gene percentage (percent.mt) < 10%, and total unique molecular identifier count (nCount_RNA) < 50,000 (Hao et al., 2021). For the filtered data, to reduce dimensionality, the top 2,000 HVGs were selected using the vst method based on their high coefficient of variation. The top 10 genes from this set were then highlighted using the LabelPoints function. Subsequently, the ScaleData function was used to normalize and scale all of the genes. Based on these 2,000 genes, the RunPCA function was used to perform principal component analysis (PCA), and the principal components (PCs) suitable for subsequent analyses were selected. Next, to delineate cell populations, clustering analysis was conducted with the FindNeighbors and FindClusters functions. Clustering resolution was set to 0.3, which ensured clear annotation of cell types without excessive or insufficient clustering of cell populations. For visualization, the uniform manifold approximation and projection (UMAP) algorithm was subsequently applied based on the PCs, generating plots that illustrated the distribution of cell clusters and highlighted differences across samples. To further elucidate the functional properties of each cell cluster, cell types of different clusters were annotated using marker genes from reference literature and the CellMarker database (http://bio-bigdata.hrbmu.edu.cn/CellMarker/)(Zheng et al., 2024). The DotPlot function was used to analyze gene expression levels across different cell clusters and confirm the type of each cluster. In addition, the FindAllMarkers function was used to identify marker genes significantly upregulated in each cell cluster (with thresholds of log2 FC ≥ 1 and *p* < 0.05). Following annotation of cell clusters using the top five marker genes (log2FC-ranked), intergroup differences in cell type abundance were compared using bar charts to identify differentially abundant cells. Among these, TRPM4-high cells were classified as key cells, and DEGs between the tumor and normal groups were identified using the FindMarkers function in Seurat (v5.1.0; |log2FC| > 0.25, min.pct ≥0.1). Volcano plots were generated with the “ggvolcano” package (v 0.0.2) to visualize the top 10 upregulated and downregulated genes. Concurrently, TRPM4 expression differences in key cell types were assessed using the Wilcoxon rank-sum test (*p* < 0.05) and visualized with the “ggpubr” package (v 0.6.0).

### 2.4 Identification of module genes in BLCA key cells using hdWGCNA

We used the GSE222315 dataset and the “hdWGCNA” package (v0.4.00) to construct a scale-free network, as previously described (Morabito et al., 2023), to identify module genes associated with key BLCA cell types.

First, the SetupForWGCNA function was used to select genes with expression proportions above 5% for inclusion in the analysis, thereby ensuring a minimum level of gene expression universality. Subsequently, cells were grouped by cell_type and orig.ident, and metacells were constructed in the harmony dimensionality-reduction space using the k-nearest neighbors (KNN) algorithm (k = 25). The metacell expression matrix was then standardized to obtain a high-quality data foundation for the subsequent network construction. The TestSoftPowers function screened soft-thresholding powers (β) from 1 to 30 to identify a value that achieved a scale-free fit index (*R* ^2^) of at least 0.8, thereby meeting the scale-free network criterion. The ConstructNetwork function was invoked with “key cells” as the network identifier to build a coexpression network from the unsigned topological overlap matrix. The PlotDendrogram function was used to construct a dendrogram of module clustering, visually displaying the relationships among modules. In the module gene analysis, module genes were ranked by calculating key module eigengene-based connectivity (kME). The top 10 genes with the highest kME values in each module were extracted. Assignment tables for all of the non-grey modules were generated, with module information recorded for each gene. Module gene expression was visualized with the ModuleFeaturePlot function. Subsequently, cell-level module scores were derived from these genes using the “Seurat” package (v5.1.0) and the UCell scoring method. The GetModules function was used to extract all of the genes from these key modules, ultimately yielding the key cell module genes.

### 2.5 Analyses of differentiation trajectory, cell-cell interactions, and transcriptional regulation in BLCA

To delineate the differentiation trajectories of key cell populations, we first applied dimensionality reduction and clustering as described in Section 2.3. Subsequently, we performed a pseudotime analysis using the “Monocle2” package (v2.28.0) to reconstruct the developmental paths (Qiu et al., 2017). A CellDataSet object was constructed from the gene expression matrix and metadata. Genes were identified using the differentialGeneTest function with a q-value of 0.05 and use_for_ordering set to TRUE. Independent component analysis was applied for dimensionality reduction, with a maximum of 2 components. The pseudotime trajectory was ordered and visualized using the orderCells function, and a tree-like structure of cell-state transitions was generated. Significant pseudotime-related genes were identified using the differentialGeneTest function with a q-value of 0.01 and use_for_ordering set to TRUE. TRPM4 expression was mapped onto the pseudotime trajectory for visualization.

Single-cell transcriptomic data were analyzed using “CellChat” (v1.6.1) to comprehensively characterize the interaction patterns between key cells and other cell types (Huang et al., 2024). The human secreted signaling pathway database was used as the reference for ligand-receptor interactions. Significantly overexpressed ligands and receptors were first screened for each cell type and subsequently mapped onto a protein–protein interaction network. Centrality analysis was performed to elucidate the functional differences among cell subpopulations in terms of information senders and receivers. Visualization analysis was further conducted on biologically significant signaling pathways to assess the involved ligand-receptor pairs.

To investigate differences in transcriptional regulatory networks between BLCA and normal cells, we analyzed a single-cell dataset using the Single-Cell Regulatory Network Inference and Clustering (SCENIC) workflow. The normalized expression matrix was extracted from the “Seurat” package (v5.1.0). After gene filtering (genes expressed in at least 1% of cells and with a total expression count of ≥3), the Gene Expression and Network Inference with Ensemble of Trees algorithm was used in combination with the RcisTarget database to construct the transcriptional regulatory network. Active transcription factors (TFs) with an average area under the curve (AUC) > 0.03 were identified using the AUCell scoring method. Differential TF analysis between tumor and normal groups was performed using the FindMarkers function in the “Seurat” package (v5.1.0), with a significance threshold of p.adj <0.01. In addition, TFs associated with TRPM4 were extracted from SCENIC and intersected with the differential expression results to identify significant TRPM4 regulatory TFs. The top 20 significant TFs were visualized in a bubble plot.

### 2.6 Identification of prognostic genes

To obtain candidate genes, we determined the intersection of DEGs1, DEGs2, and key cell module genes using the “ggvenn” package (v 0.1.9).

To identify prognostic genes, we first performed univariate Cox regression analysis (coxph function; “survival” package v3.7.0) on candidate genes, applying thresholds of HR ≠ 1 and *p* < 0.05. Following initial screening, the proportional hazards (PH) assumption test (*p* > 0.05) was applied to exclude false positives. Finally, multivariate Cox regression was conducted to adjust for potential confounders. The step function from the “stats” package (v4.3.1) was applied to perform bidirectional stepwise regression for variable selection, and a risk score formula was constructed. The overall model was tested for PH assumptions using cox.zph to screen for final prognostic genes (*p* > 0.05), as follows:

$$\text{Risk score}=\sum_{i=1}^{n}\text{coef }\left({\text{gene}}_{i}\right)\times \text{expr }\left({\text{gene}}_{i}\right),$$

where coef and expr denote the risk coefficient and gene expression level, respectively.

### 2.7 Construction and validation of a risk model

The median risk score from the TCGA-BLCA training dataset was used as the cutoff to assess the association between risk scores and patient survival. This approach dichotomized the 404 patients with BLCA into distinct high-risk (HRG) and low-risk (LRG) groups. The minprop parameter was set to ensure that each group’s proportion was no less than 30% of the total. The “survminer” package (v 0.4.9) was applied to visualize the risk score distribution and survival status (Liu et al., 2021). Concurrently, the “pheatmap” package (v1.0.12) was used to generate heatmaps depicting prognostic gene expression. K–M curves were plotted (“survminer” package) and compared using the log-rank test to assess the survival difference and evaluate survival differences (*p* < 0.05). Time-dependent receiver operating characteristic (ROC) curves for 1, 3, and 5 years were generated to assess predictive performance (“timeROC” v0.4), retaining models with an AUC in the range of 0.6–1 (Lin et al., 2023). The model’s generalizability was then tested by validating it in the external GSE13507 cohort using identical criteria and methods. To further evaluate the reliability of the nomogram, we used ROC curves to assess the discriminatory performance of models incorporating only independent clinical prognostic factors, models incorporating only prognostic genetic risk scores, and the nomogram model in the training set, calculating the AUC values for 1-year, 3-year, and 5-year follow-up.

### 2.8 Nomogram construction and validation for BLCA prognostic risk assessment

To screen for independent risk factors, in the training set TCGA-BLCA, univariate Cox regression analysis (HR ≠ 1, *p* < 0.05) and the PH assumption test (*p* > 0.05) were applied to evaluate clinical characteristics (e.g., age, sex, TNM stage, pathological stage) and risk scores using the “survival” package (v3.7.0). Subsequently, a multivariate Cox model was built using PH-compliant clinical characteristics. This initial model, fitted with coxph, was refined via akaike information criterion-based bidirectional stepwise regression (“stats” package v4.3.1) and subsequently validated for PH compliance (*p* > 0.05). The prediction model was presented as a nomogram using the “regplot” package (v1.1) and subsequently validated for reliability and clinical utility. Calibration curves (1/3/5-year) from the “rms” package (v6.8-1) and decision curves from the “ggDCA” package (v1.1) were used for these assessments, respectively (Zhang et al., 2024). In addition, prognostic genes were matched with patients’ follow-up information. We screened for prognostic genes by evaluating the correlation between each gene’s expression level and survival outcomes using univariate Cox regression models. For protective factors with a regression coefficient <0, expression values were inverted in subsequent analyses to unify the direction of risk prediction. Subsequently, time-dependent ROC analysis at 1, 3, and 5 years was performed using the “timeROC” package (v0.4) to validate the nomogram’s performance, requiring an AUC between 0.7 and 1.

### 2.9 GSEA and immune microenvironment analysis

Gene set enrichment analysis (GSEA) was performed using the “clusterProfiler” package (v4.2.2) to assess predictive performance (Wu et al., 2021). The analysis employed a pre-ranked gene list (sorted by log2FC from “DESeq2” v1.4.2 comparison of HRG vs. LRG) and the c2.cp.kegg.v7.4.symbols.gmt dataset from Molecular Signatures Database, with significance criteria of |NES| > 1, *p* < 0.05, and *q* < 0.25.

To characterize the immune landscape, we used the CIBERSORT algorithm to quantify 22 immune cell types in both groups. The Wilcoxon test (*p* < 0.05) was used to identify significant differences, and results were visualized with “ggplot2” (v3.4.1). To investigate immune regulatory networks, the “psych” package (v2.1.6) was used to analyze correlations (|cor| > 0.3, *p* < 0.05) involving immune cells and prognostic genes (Robles-Jimenez et al., 2021).

### 2.10 Drug sensitivity and prognostic gene protein verification in BLCA

To investigate the therapeutic effects of chemotherapeutic drugs on patients with BLCA, we used the built-in dataset cgp2016 of the “pRRophetic” package (v0.5) to screen drugs with TCGA.classification recorded as BLCA and AUC >0.98 (Geeleher et al., 2014). To predict chemotherapeutic responses, we estimated drug IC50 values in each risk group using the pRRopheticPredict function (tissueType = “urogenital_system”). The Wilcoxon rank-sum test was applied to compare group differences *(p* < 0.05). Spearman correlations between prognostic genes and the top 50 differentially effective drugs were calculated to explore the underlying mechanisms involved (|cor| > 0.3, *p* < 0.05).

To validate the protein expression of prognostic genes, immunohistochemistry (IHC) data for BLCA were retrieved from the Human Protein Atlas (HPA) database (https://www.proteinatlas.org/) and analyzed for staining intensity, the proportion of positive cells, and subcellular localization patterns in BLCA tissues.

### 2.11 Quantitative real-time polymerase chain reaction (qRT–PCR)

This study procured tumor tissues and their paired normal adjacent tissues from 10 patients diagnosed with BLCA who were admitted to the First Hospital of Lanzhou University in 2024. Following excision, all specimens were immediately frozen in liquid nitrogen and subsequently stored in a −80 °C freezer. Comprehensive clinical and pathological data pertaining to the patients, including age, sex, tumor grade, and pathological stage, is delineated in Supplementary Table S1. This study was approved by the Ethics Committee of the First Hospital of Lanzhou University (Approval No. LDYYLL-2025-2073), and all patients provided written informed consent. Total RNA was extracted using TRIzol reagent (Vazyme, China), and qualified samples (A260/A280: 1.8–2.0, NanoDrop 2000) were subsequently analyzed by qRT–PCR. Reactions were performed in 10 µL volumes comprising 5 µL ChamQ Blue SYBR qPCR Master Mix (Vazyme, China), 0.4 µL primers, 1 µL cDNA, and 3.6 µL water, using a QuantStudioTM3 Flex System (Applied Biosystems, United States) under the following conditions: 95 °C for 30 s, followed by 40 cycles of 95 °C for 5 s and 60 °C for 30 s. For each sample, three technical replicates were established, and the mean Ct value was utilised for analysis. GAPDH was utilised as the internal reference gene, and the relative expression levels of the target genes were calculated using the 2^(-ΔΔCt) method. It is imperative to note that all experiments utilised 10 independent biological samples, thus ensuring the reliability of the results obtained. All of the primer sequences are provided in Supplementary Table S1, and data were visualized with GraphPad Prism (v9.5.0).

### 2.12 Statistical analysis

Data analysis was performed in R (v4.2.2) using the Wilcoxon test for two-group comparisons, except for qRT–PCR data, which was analyzed using a *t*-test, with significance defined as *p* < 0.05.

## 3 Results

### 3.1 TRPM4-related DEGs and survival outcomes in BLCA

**FIGURE 1:**
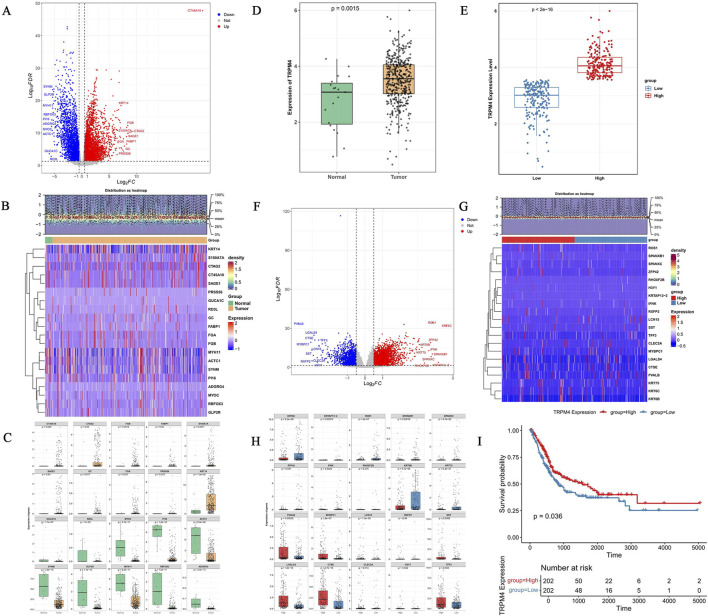
Analysis of DEGs, differential expression of TRPM4, and survival in BLCA patients in the TCGA-BLCA. **(A)** Volcano plot depicting bladder cancer DEGs and sample hierarchical clustering. **(B)** Heatmap of the up- and downregulated DEGs. **(C)** Box plots showing the differential infiltration landscape of the top 10 genes in bladder cancer and normal samples. **(D)** The expression of TRPM4 in bladder cancer and normal samples. **(E)** Boxplot showing that high-expression patients have significantly higher TRPM4 expression levels than low-expression patients. **(F)** Volcano plot depicting DEGs and sample hierarchical clustering. **(G)** Heatmap of DEGs2 from the high-and low-expression groups of TRPM4. **(H)** The box plot showing the expression of the top 10 up-and downregulated genes. **(I)** The Kaplan-Meier survival curve of the generation probability of patients in the low and high-expression groups. The prognosis of the patients in the high expression group is significantly higher than that of the low expression group; the difference is statistically significant.

A total of 7,808 DEGs1 were identified in the TCGA-BLCA training set analysis of the tumor versus normal groups (4,714 upregulated, 3,094 downregulated; |log2FC| > 0.5, *p* adj <0.05), displayed in a volcano plot with color-coding (red for upregulated (e.g., FGB), blue for downregulated (e.g., MYOC), and gray for non-significant genes; Figure 1A). A heatmap was constructed to show the expression pattern of DEGs1 across different samples, along with a density plot showing the frequency distribution of DEGs1 expression levels (Figure 1B). The top 10 upregulated and downregulated genes all exhibited significant differential expression between the normal and tumor groups (*p* < 0.05) (Figure 1C). TRPM4 expression was significantly upregulated in BLCA tumors (*p* = 0.0015), indicating its potential promoting effect on the oncogenesis and development of this malignancy (Figure 1D). The high-TRPM4 expression group exhibited significantly elevated TRPM4 levels (*p* < 2 × 10^−16^, Figure 1E) and a corresponding transcriptomic alteration of 4,683 DEGs2 (3,106 upregulated, 1,577 downregulated; |log2FC| > 0.5, *p*.adj <0.05). The distribution of these genes was visualized in a volcano plot (red dots: upregulated genes (e.g., ZEP42); blue: downregulated genes (e.g., SST); gray: non-significant genes; Figure 1F). A heatmap was constructed to show the expression patterns of DEGs2 across different samples, along with a density plot showing the frequency distribution of their expression levels (Figure 1G). The top 10 upregulated and downregulated genes were not only differentially expressed (*p* < 0.05; Figure 1H) but also showed prognostic relevance, with high expression conferring a significant survival advantage (*p* = 0.036) (Figure 1I) (Supplementary Table S2).

### 3.2 ScRNA-seq analysis of tumor and normal: epithelial cells and TRPM4

**FIGURE 2:**
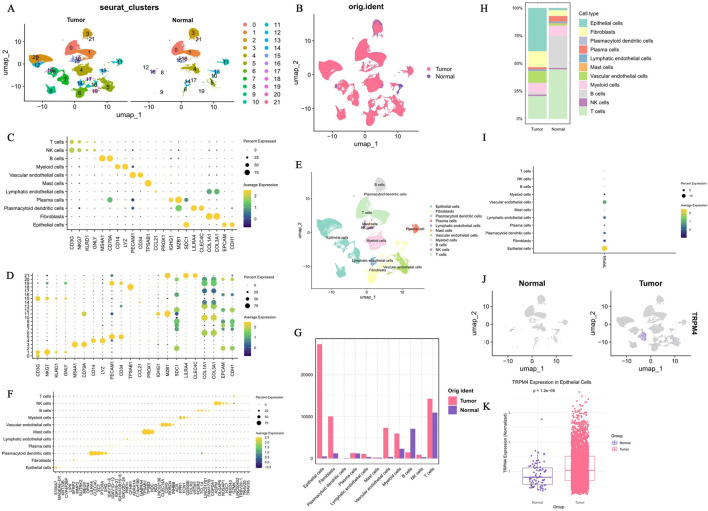
Analysis of the BLCA Single-Cell Dataset GSE222315 and Expression Characteristics of TRPM4. **(A,B)** Single-cell UMAP analysis reveals transcriptional landscape and cellular heterogeneity in bladder cancer and normal tissue samples. Display respectively by cell clusters **(A)** and sample sources **(B)**. **(C,D)** Bubble plot displaying gene expression patterns across major cell types. **(E)** UMAP projection of single cells colored by cell type identity. **(F)** The top 5 characteristic genes of each cell subpopulation. **(G)** The differences in the quantities of different cell subpopulations in BLCA and NAT. **(H)** The proportion of different cell subgroups in BLCA and NAT. **(I)** TRPM4 exhibits the highest expression in epithelial cells. **(J)** The distribution of expression of the TMPR4 gene is examined at the level of individual cells. **(K)** TRPM4 Expression in Epithelial Cells of the BLCA and NAT.

First, quality control processing was performed on the raw data of the single-cell dataset GSE222315 to facilitate subsequent analyses. Before quality control, the initial cell counts were 102,226, and the gene counts were 24,739. After quality control, the cell counts were optimized to 94,425, whereas the gene counts remained unchanged at 24,739 (Supplementary Figure S1A,B). Subsequently, the top 2,000 HVGs were identified, among which the top 10 most variable genes included IGKV3-11, IGKV3-20, and IGKC (Supplementary Figure S1C). The curve flattened at PC = 25; thus, the first 25 PCs were selected for further analysis (Supplementary Figure S1D,E). Next, the cells were clustered into 22 clusters using UMAP. After batch correction, the distribution of cell clusters was primarily driven by biological differences, eliminating batch bias due to sample origin (Figures 2A,B). These 22 cell clusters were annotated as 11 cell types: epithelial cells, T cells, natural killer cells (NK), B cells, plasma cells, fibroblasts, vascular endothelial cells, myeloid cells, mast cells, lymphatic endothelial cells, and plasmacytoid dendritic cells (Figures 2C–E). T cells showed high GZMK gene expression, and the ASPM gene was similarly highly expressed in NK cells, reflecting molecular differences among immune cells (Figure 2F). Compared with normal tissues, the tumor microenvironment showed markedly elevated abundances of epithelial cells and T cells, along with significant differences in the proportional distribution of major cell types, including B cells (Figures 2G,H). Further, TRPM4 was significantly highly expressed in epithelial cells; thus, epithelial cells were considered key cells (Figures 2I,J). A total of 1,346 genes differentially expressed in epithelial cells were ultimately identified (Supplementary Figure S1F). Meanwhile, TRPM4 expression showed a significant difference in epithelial cells between tumor and normal groups (*p* = 1.2 × 10^−5^) (Figure 2K) (Supplementary Table S3).

### 3.3 Gene coexpression analysis identifies turquoise module linked to BLCA/normal and epithelial cell function

**FIGURE 3:**
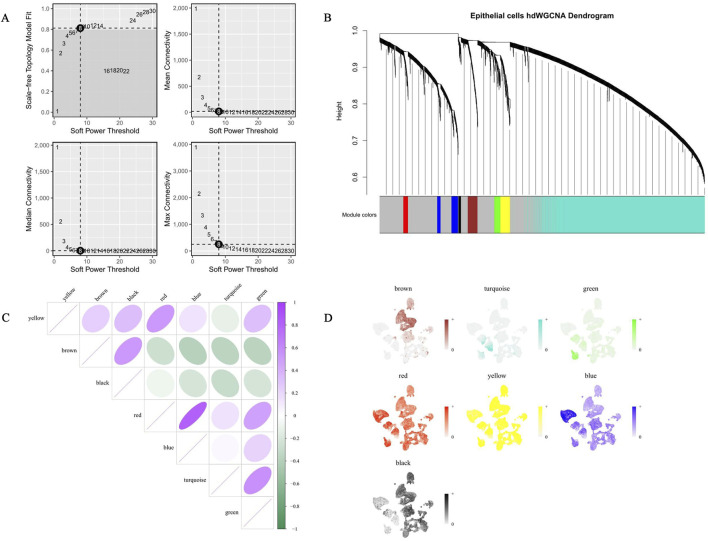
Identification and characteristic analysis of co-expression modules related to key BLCA cells based on hdWGCNA. **(A)** Network topology analysis under varying soft power thresholds. **(B)** Hierarchical clustering dendrogram of epithelial cells using hdWGCNA analysis. **(C)** Heatmap visualization of correlation patterns among co-expression modules. **(D)** Spatial expression patterns of seven co-expression modules identified through hdWGCNA analysis.

When R2 = 0.8, the soft power threshold was set to 8 (Figure 3A). Subsequently, using this soft threshold, a clustering tree was constructed, yielding a total of seven coexpression modules after excluding the grey module (Figure 3B). Meanwhile, the distribution of the top 10 module genes ranked by kME values in these seven modules was obtained. In addition, the yellow and red modules showed a deep purple color, indicating a strong positive correlation between them and suggesting that the modules were highly coordinated in terms of gene coexpression patterns (Figure 3C). Further, the turquoise module was predominantly enriched in epithelial cells, and its expression profile was highly consistent with that of the TRPM4 gene, suggesting that this module may dominate the functional state of epithelial cells (Figure 3D). The turquoise module contained 4,802 genes, defined as key cell module genes (Supplementary Table S4).

### 3.4 Epithelial cell subclusters and TRPM4’s role in the differentiation trajectory

**FIGURE 4:**
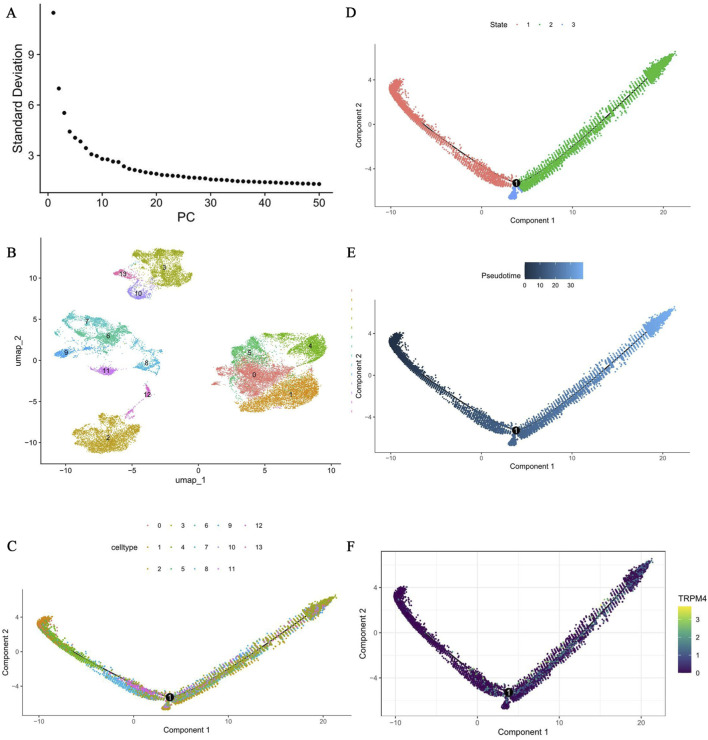
Subclustering of BLCA epithelial cells and expression characteristics of TRPM4 in the cell differentiation trajectory. **(A,B)** Perform secondary clustering analysis on epithelial cells. **(C,D)** Pseudo-temporal analysis of key gene sets based on cell types **(C)** and developmental time **(D)**. **(E)** Pseudo-temporal analysis of key gene sets based on pseudo-temporal time. **(F)** Differential expression analysis of whole-genome expression data based on pseudo-temporal sequencing.

Further secondary dimensionality reduction and clustering analysis of the epithelial cells yielded 14 distinct subclusters (Figures 4A,B). In the pseudotime analysis, the epithelial cells followed a continuous, progressive differentiation trajectory, potentially reflecting the differentiation stages of each cell subcluster. These 14 subclusters could be roughly divided into 3 differentiation stages (Figures 4C,D). Specifically, the pseudotemporal analysis clearly showed the progressive trajectory of epithelial cells, from an early state (dark blue) to a more mature state (light blue), providing important clues for analyzing the potential transition path of epithelial cells (Figure 4E). During differentiation, TRPM4 showed a significant expression gradient along the pseudotime, with high expression mainly concentrated in the late developmental stage. This spatiotemporal expression pattern implies that TRPM4 may play a key regulatory role during epithelial cell state transitions (Figure 4F).

### 3.5 Transcriptional regulation and cell communication analyses of epithelial cells

**FIGURE 5:**
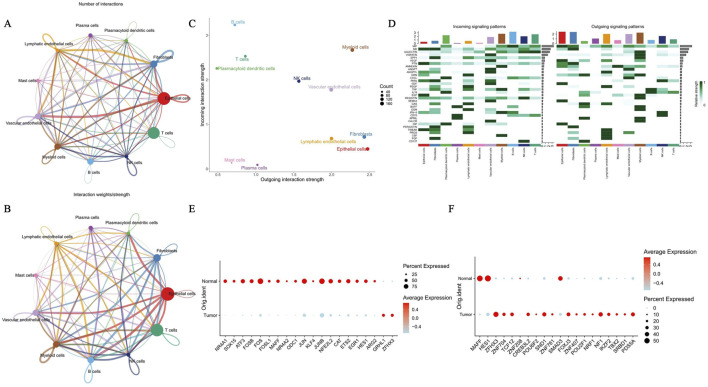
Analysis of cell communication patterns (MIF/MK pathways) and transcriptional regulatory networks in BLCA epithelial cells. **(A,B)** Based on cellular communication network analysis, epithelial cells demonstrate strong cellular communication capabilities. Analyze the cellular network through both the number **(A)** and the weight of cell interactions **(B)**. **(C,D)** Cell-cell interaction strength analysis suggests that epithelial cells serve as key contributors and primarily mediate communication through the MIF and MK pathways. Scatter plot of intercellular interaction strength distribution **(C)**. Heatmap of Intercellular Signaling Pathways **(D)**. Note: The left side shows the incoming signaling patterns, while the right side displays the outgoing signaling patterns. **(E)** Analysis of differentially expressed regulatory TFs in normal and tumor tissues, including a bubble chart of the top 20. **(F)** Analysis of differentially expressed regulatory TFs associated with TRPM4 in normal and tumor tissues, including the top 20 bubble chart.

In the cell communication analysis, the epithelial cell type exhibited greater network centrality and thicker connections with vascular endothelial cells and myeloid cells across the two dimensions of interaction count and interaction weight. This finding indicates a large number of interactions between these cells and a higher interaction strength, suggesting that these cells play a crucial role in intercellular communication (Figures 5A,B). Epithelial cells served as signal producers, contributed to cell communication, and primarily exerted their main communication function through the macrophage migration inhibitory factor (MIF) and midkine (MK) pathways (Figures 5C,D). Three primary ligand–receptor interaction patterns were detected in the MIF pathway: MIF–ACKR3, MIF–(CD74 + CXCR4), and MIF–(CD74 + CD44). In the MK (MDK) pathway, the detected receptor combinations were more diverse, primarily MDK–SDC4, MDK–SDC2, MDK–SDC1, MDK–NCL, MDK–LRP1, and MDK–(ITGA6 + ITGB1) (Supplementary Figures S2A,B). Analysis of the epithelial transcriptional network identified FOS and JUNB as key factors with reduced expression in tumor tissues compared with normal epithelia. The tumor group showed distinct expression patterns. When screening for upstream factors regulating TRPM4 expression, the top 20 differentially expressed TFs were identified between tumor and normal groups. A marked contrast was observed in MAFF, which was highly expressed in the normal group but significantly downregulated in tumor tissues (Figures 5E,F).

### 3.6 Construction and validation of a prognostic model

**FIGURE 6:**
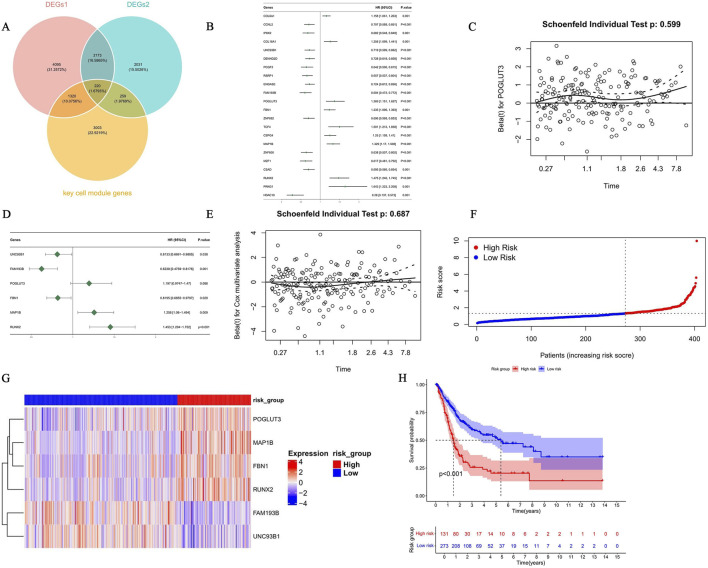
A bladder cancer risk model was constructed and validated based on six selected prognostic genes. **(A)** Venn diagram analysis of three sets of gene overlap relationships. **(B)** Forest plot of the association between 22 candidate genes selected by COX regression analysis based on the aforementioned candidate genes and clinical outcomes. **(C)** Using the multivariate COX regression model for further analysis of prognostic genes, a forest plot of 22 candidate genes was obtained. **(D)** The multivariate Cox model satisfies the PH test. **(E–G)** Risk curve **(E)**, survival analysis **(F)**, and gene expression **(G)** analysis between HRGs and LRGs based on the risk scoring model constructed from the aforementioned candidate genes. **(H)** Kaplan-Meier survival analysis shows that the overall survival rate in the HRG is significantly lower.

The intersection of 7,808 DEGs1, 4,683 DEGs2, and 4,802 key cell module genes was determined, yielding 220 candidate genes (Figure 6A) (Supplementary Table S5).

To refine the prognostic gene set, the 220 initial candidates were screened in the training set using univariate Cox regression (*p* < 0.05, HR ≠ 1) and the PH assumption test (*p* > 0.05), resulting in 22 candidate genes. Among them, genes with HR greater than 1 were considered risk factors, including COL6A1, COL18A1, POGLUT3, FBN1, TCF4, CSPG4, MAP1B, RUNX2, and PRKG1, indicating that high expression of these genes was significantly associated with poor patient prognosis. In contrast, genes with HR below 1 were considered protective factors, such as CCNL2, IP6K2, UNC93B1, DENND2D, PCGF3, RSRP1, ENGASE, FAM193B, ZNF600, MZF1, CSAD, and HDAC10 (Figure 6B). To further refine the model, we combined multivariate Cox regression with bidirectional stepwise regression for PH-compliant genes, followed by a PH test to verify the final model’s validity, screening six prognostic genes: UNC93B1, FAM193B, POGLUT3, FBN1, MAP1B, and RUNX2 (Figures 6C,D) (Table 1).

**TABLE 1:** Results of univariate Cox regression analysis.

Id	HR	L95CI	H95CI	pvalue	PH_pvalue
COL6A1	1.15757864261593	1.06109752264289	1.26283238368421	0.000982226126832787	0.543082825854692
CCNL2	0.707145141558875	0.58791189585277	0.850559845374432	0.000235118730278191	0.106009117186215
IP6K2	0.681531007246765	0.547875683015164	0.847791804305967	0.000576345546001914	0.260427710231443
COL18A1	1.25820905952989	1.09868684651966	1.44089286451174	0.00089831318081957	0.928964025365801
UNC93B1	0.718886048571431	0.599312677125533	0.86231640103017	0.00037665847195209	0.118822883320664
DENND2D	0.728182535540082	0.617813624132441	0.858268229047523	0.000155271799233601	0.204359073179177
PCGF3	0.642023336115805	0.505689760747461	0.815112339842523	0.000274206462480394	0.0946294490991199
RSRP1	0.65665296548796	0.53651924490555	0.803686207304716	4.505211109545e-05	0.110719268248345
ENGASE	0.72356494979513	0.61173191243724	0.855842610018718	0.000158608071163087	0.0788910634037
FAM193B	0.603590321407565	0.472050975616151	0.771783758356504	5.68508089834966e-05	0.751124305978884
POGLUT3	1.36854653114473	1.15103442089091	1.62716211949476	0.000381242724474591	0.599410585105639
FBN1	1.23537175548909	1.09589624219034	1.39259841899808	0.000543948952749528	0.540071384425968
ZNF692	0.696000544621535	0.568074659961121	0.852734318666189	0.00046993027367854	0.0639294492683811
TCF4	1.50103019599172	1.21280467547948	1.85775310306104	0.000188835733560324	0.475004791633913
CSPG4	1.24998352897295	1.1077891008509	1.41042985664287	0.00029308607165069	0.592466591997564
MAP1B	1.328715	1.17040176354137	1.50844194412959	1.12908459586827e-05	0.969587685854584
ZNF600	0.63763303500363	0.507184423333913	0.801633229694566	0.000116549557945046	0.11559830653126
MZF1	0.617145727563351	0.480616072953918	0.792459658514617	0.000154727793598217	0.132486831082354
CSAD	0.695115046769914	0.565979650199714	0.853714313006556	0.000523826539150914	0.379453143237311
RUNX2	1.47460484266312	1.24621727838925	1.74484777230504	6.07769778739322e-06	0.664001966063033
PRKG1	1.64348344934033	1.22331599768007	2.20796413467814	0.000973742525998622	0.423116424992605
HDAC10	0.279888174664714	0.136618542525256	0.573402327891645	0.000501667969517396	0.372200417092681

Subsequently, a prognostic model was constructed with these six prognostic genes. The 404 eligible patients with BLCA were categorized into 131 HRG and 273 LRG patients, according to the optimal risk score cutoff of 1.323915. The risk score distribution analysis revealed an ascending gradient from left to right, paralleled by an increasing number of patient deaths. The distribution of survival outcomes indicated that surviving patients were mostly concentrated in the LRG, whereas deceased patients were mostly concentrated in the HRG (Figures 6E,F). The six-gene risk signature stratified patients into the HRG and LRG, with lower gene expression conferring lower risk (Figure 6G). This stratification predicted significant survival differences (*p* < 0.001; Figure 6H) and demonstrated good prognostic accuracy over 1–5 years (AUCs: 0.65, 0.68, 0.67; Supplementary Figure S3A). Similarly, applying the optimal cutoff (1.617068) to the GSE13507 validation cohort (n = 165) stratified patients into the HRG (n = 65) and LRG (n = 100). All of the key findings from the training set were successfully replicated, suggesting the robustness of the risk model (Supplementary Figure S3B,F). In summary, the prognostic model demonstrated stable, reliable predictive performance.

### 3.7 A nomogram for prognosis based on independent factors

**FIGURE 7:**
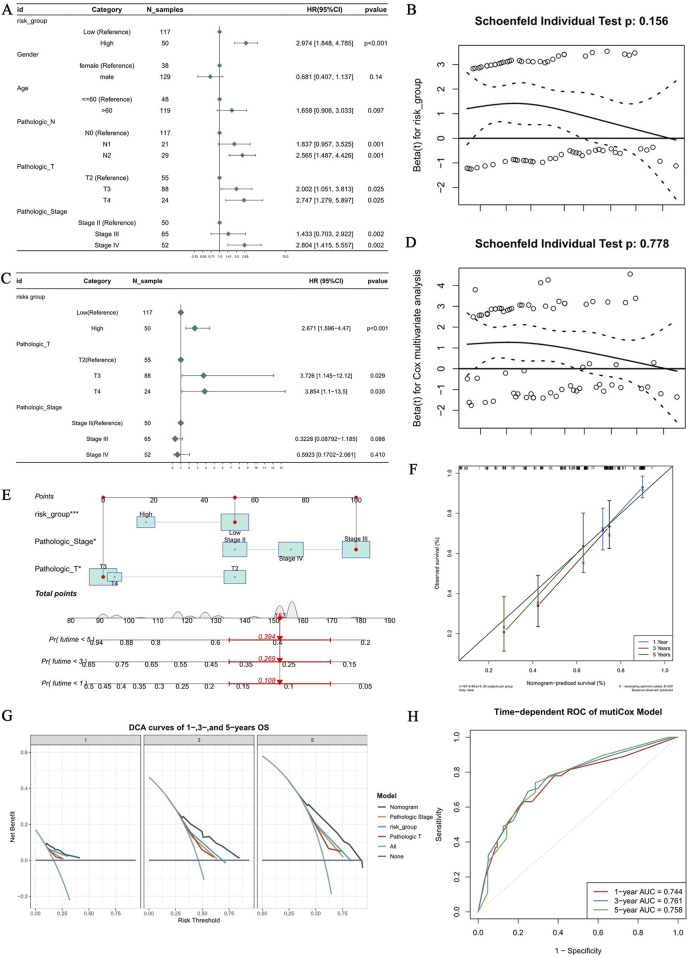
Develop and validate a BLCA prognostic nomogram using independent prognostic factors. **(A)** Forest plot showing univariate Cox regression analysis of the association between clinicopathological characteristics and overall survival. Hazard ratios (HR) with 95% confidence intervals (CI) are presented for each variable. **(B)** Conduct a PH test for the risk group. **(C)** Forest plots suggest that risk groups, pathological T-staging, and pathological stages can serve as independent prognostic factors. **(D)** Multivariate Cox analysis satisfies the PH test. **(E)** A nomogram for predicting 1-, 3-, and 5-year overall survival. **(F)** Calibration curve of predicted survival rate by nomogram versus actual observed survival rate. **(G)** Decision Curve Analysis (DCA) for 1-year, 3-year, and 5-year Overall Survival Rates (OS). **(H)** Time-dependent ROC curves for the multi-variable Cox regression model at 1, 3, and 5 years. The ROC curve suggests that the model has good predictive ability.

Univariate Cox regression identified risk_group, Pathologic_Stage, Pathologic_N, and Pathologic_T as significant prognostic factors (HR ≠ 1, *p* < 0.05; Figure 7A), and this model satisfied the PH assumption (*p* > 0.05; Figure 7B). A multivariate model confirmed that risk_group, Pathologic_T, and Pathologic_Stage are independent prognostic factors (PH assumption met; Figures 7C,D), and the model was subsequently translated into a nomogram for predicting 1-, 3-, and 5-year survival probabilities (Figure 7E). The model demonstrated that higher total scores were associated with poorer survival outcomes, with a score of 157 indicating a notably reduced post-diagnosis survival rate. Calibration curves for all three time points were closely aligned with the reference line, confirming the high predictive accuracy of the nomogram (Figure 7F). The nomogram demonstrated substantial clinical utility, supported by high net benefits on decision curve analysis (Figure 7G) and consistent predictive power evidenced by time-dependent AUCs (0.744, 0.761, 0.758) at 1, 3, and 5 years (Figure 7H; Supplementary Table S6). In addition, the 1-, 3-, and 5-year AUC values for models that included only independent prognostic factors and the 6-gene prognostic risk score were all lower than the AUC value for the nomogram (Supplementary Figure S4).

### 3.8 GSEA pathway enrichment and immune cell infiltration differences between HRG and LRG

**FIGURE 8:**
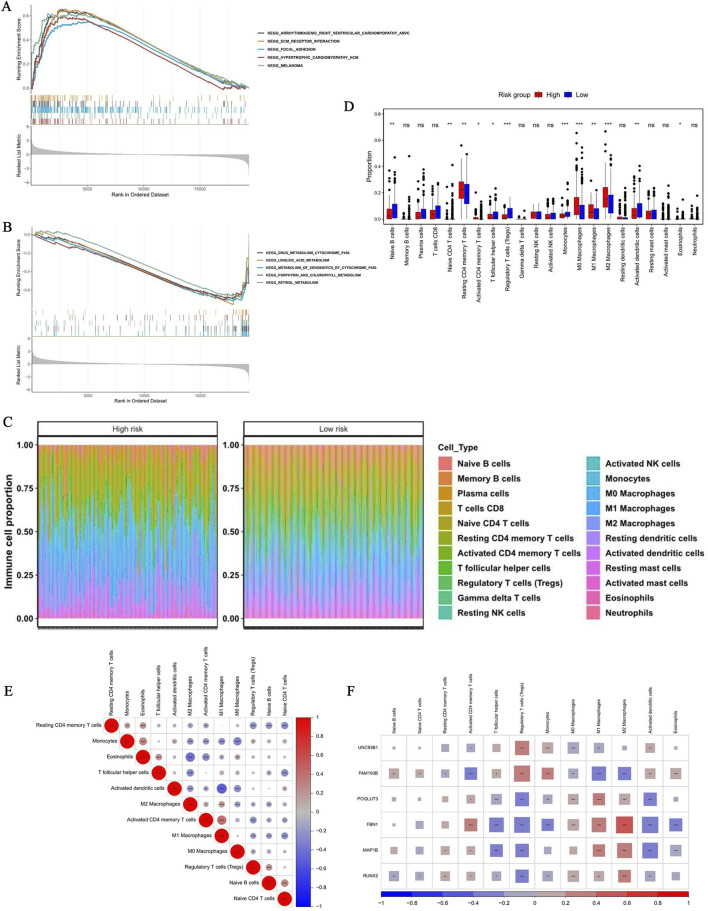
Analysis of GSEA pathways, immune infiltration, and HRG-LRG correlations in BLCA. **(A,B)** GSEA was performed to analyze the differential gene expression between HRGs and LRGs. The results showed the TOP5 significantly enriched pathways and significantly suppressed pathways in the HRG. **(C)** Immune cell infiltration profiles in HRGs and LRGs. **(D)** Comparison of immune cell infiltration levels between HRGs and LRGs. The box plots display the distribution of estimated proportions for each tumor-infiltrating immune cell type. **(E)** Heatmap of correlations between immune cell types. The results show significant interaction networks among immune cells. **(F)** Correlation analysis between immune cell abundance and gene expression levels.

GSEA revealed 47 significantly altered pathways between the risk groups, among which extracellular matrix-receptor interaction, arrhythmogenic right ventricular cardiomyopathy (ARVC), focal adhesion, melanoma, and HCM were the top five pathways activated in the HRG (Figure 8A). Pathway analysis also revealed significant inhibition of five metabolic processes in the HRG, including xenobiotic metabolism by cytochrome P450 and retinol metabolism (Figure 8B). The concerted activation of matrix-related pathways and suppression of metabolic processes offer potential mechanistic clues for the adverse prognosis associated with high risk (Supplementary Table S7).

Immune infiltration analysis revealed generally high levels of activated NK cells and monocytes across both risk groups (Figure 8C). Specifically, 12 immune cell subtypes exhibited significant intergroup differences (*p* < 0.05), including naive B cells, multiple T cell subsets (naive CD4, resting/activated CD4 memory, T follicular helper, and Tregs), macrophages (M0, M1, and M2), monocytes, activated dendritic cells, and eosinophils (Figure 8D). The analysis revealed a significant interaction network among immune cells. For example, M1 macrophages showed significant correlations with activated CD4 memory T cells and activated dendritic cells (cor >0.3, *p* < 0.05; Figure 8E). At the gene level, FBN1 demonstrated the strongest positive correlation with M2 macrophages (r = 0.42), whereas Treg cells were significantly correlated with all of the key prognostic genes (*p* < 0.05; Figure 8F).

### 3.9 Drug sensitivity and immunohistochemical findings in the BLCA HRG and LRG

**FIGURE 9:**
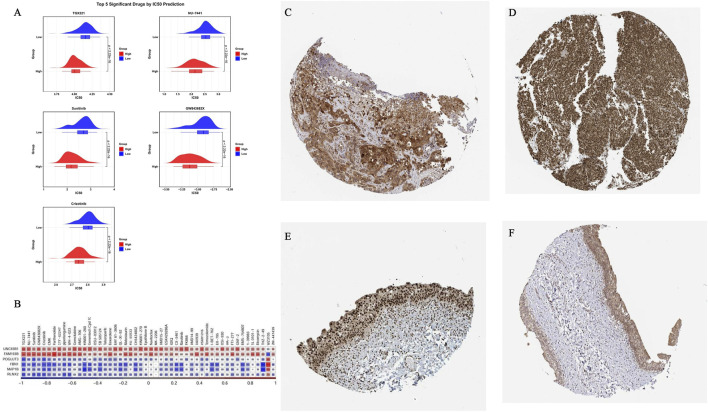
Contrast drug sensitivity (IC50) between HRGs and LRGs BLCA, and immunohistochemical validation of UNC93B1 and FAM193B. **(A)** Drug sensitivity analysis of the top five most significant drugs between HRGs and LRGs based on IC50 prediction. **(B)** Heatmap of the top 50 IC50 correlations between all key genes in the risk prediction model and therapeutic drugs. **(C–F)** Immunohistochemical results of risk model-related genes in bladder tumors and normal tissues from the HPA database. Immunohistochemical results of FAM193B in normal tissue **(C)**. Immunohistochemical results of FAM193B in tumor tissue **(D)**. Immunohistochemical results of UNC93B1 in normal tissue **(E)**. Immunohistochemical results of UNC93B1 in tumor tissue **(F)**.

Drug sensitivity analysis revealed that 112 of 175 tested compounds showed significantly different IC50 values between the HRG and LRG (*p* < 0.05). A significant positive correlation was observed between POGLUT3 expression and the IC50 of WZ3105 (|r| > 0.3, *p* < 0.05), indicating that their expression levels tended to increase or decrease in synchrony. POGLUT3 expression was negatively correlated with that of TGX221 (|cor| > 0.3, *p* < 0.05), that is, when the expression of one increased, the expression of the other tended to decrease (Figures 9A,B) (Supplementary Table S8). IHC revealed significant UNC93B1 and FAM193B protein expression in the tumor tissues of patients with BLCA. UNC93B1 showed high-intensity positive staining, mainly localized in the cytoplasm and cell membrane structures, and distributed widely; the proportion of positive cells exceeded 75%. Thus, UNC93B1 exhibited high protein expression in BLCA tissues. The example in the figure is a tissue section from an 83-year-old female patient with high-grade urothelial carcinoma, in which strong positive brown staining signals could be observed in the tumor area (Figures 9C,D). In the tissue sample of another 82-year-old female patient with BLCA, FAM193B presented a high-intensity nuclear expression pattern: the staining intensity was evaluated as “strong,” and the proportion of positive cells also exceeded 75%, suggesting that the gene has extensive and significant nuclear localization expression characteristics in BLCA tissues (Figures 9E,F).

### 3.10 qRT–PCR validation of prognostic genes

**FIGURE 10:**
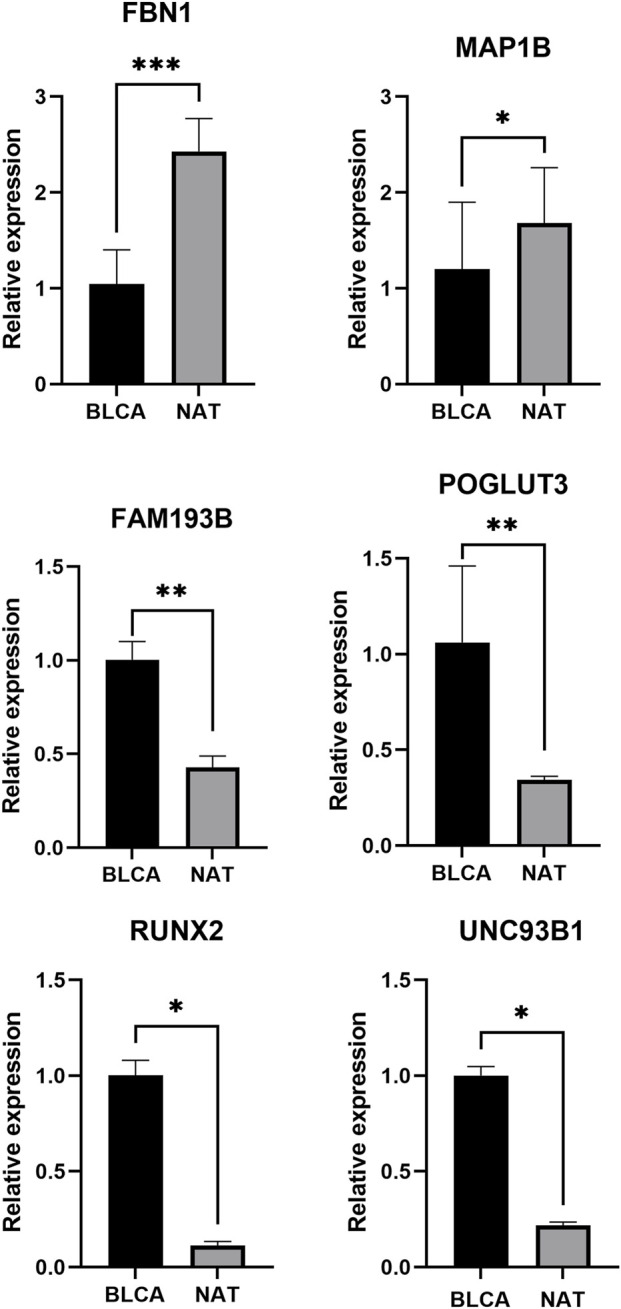
qRT–PCR validation of six prognostic genes in BLCA. Note: *: *p* < 0.05; **:*p* < 0.01; ***: *p* < 0.001.

In the TCGA-BLCA cohort, prognostic genes FBN1 and MAP1B were significantly downregulated in tumors, whereas UNC93B1, FAM193B, POGLUT3, and RUNX2 were upregulated. These expression patterns validated our bioinformatic predictions and further support their potential roles in BLCA prognosis (Figure 10).

## 4 Discussion

BLCA is a common urinary malignancy. Its pathogenesis involves multiple gene mutations and abnormal signaling activation (Aveta et al., 2022; Caballero et al., 2023), leading to uncontrolled proliferation, apoptosis suppression, and enhanced tumor invasion/metastasis. In addition, changes in the immune microenvironment significantly contribute to this process (Lin et al., 2025). However, current BLCA therapies, namely, surgery, chemotherapy, radiotherapy, and immunotherapy, have limitations, necessitating novel strategies (Kapriniotis et al., 2024). TRPM4 has attracted the attention of researchers seeking novel therapeutic targets as it is highly expressed in BLCA (vs. adjacent non-cancerous tissues), correlating with enhanced cell proliferation, migration, and invasion (Ceylan et al., 2016). Notably, TRPM4 plays a critical role in sodium overload-induced cell death (Fu et al., 2025), and its high expression in BLCA may regulate intracellular sodium homeostasis, thereby affecting cell survival/death and influencing tumor progression and prognosis (Chang et al., 2025). This study integrated single-cell transcriptomic data to investigate the role of TRPM4 in BLCA. Using TCGA-BLCA/GEO datasets, single-cell sequencing, and hdWGCNA, we analyzed TRPM4’s expression/prognosis, annotated key cells, and identified pivotal module genes. These studies yielded six prognostic genes, namely, UNC93B1, FAM193B, POGLUT3, FBN1, MAP1B, and RUNX2, for risk model construction/validation. The findings indicated that the survival rates were lower in the HRG, and the model demonstrated a satisfactory predictive capacity. Concurrently, GSEA, immune infiltration, and drug sensitivity analyses further revealed differential pathways and drug responses. IHC from HPA validated the high protein expression of prognostic genes. This study suggests elevated TRPM4 expression in BLCA, which may correlate with poor prognosis.

Single-cell analysis has revealed that epithelial cells are a distinguishing feature of BLCA. Epithelial cells are the most abundant cell type in BLCA tumors (core functional cells of the bladder urothelium, with significantly higher numbers in tumor vs. normal groups) (Huang et al., 2017). Their abnormal proliferation is a core characteristic of BLCA initiation and progression (Deng et al., 2016). EMT4—a pivotal sodium influx channel protein—shows specific expression in epithelial cells, suggesting epithelial cells as the key niche for TRPM4’s biological functions. A body of research suggests a correlation between high TRPM4 expression and prostate cancer metastasis. The study indicates that high TRPM4 expression may contribute to epithelial–mesenchymal transition (EMT) and cell migration by regulating calcium signaling (Bochen et al., 2025). In conjunction with our observations that TRPM4 is distinctly overexpressed in BLCA epithelial cells, this finding suggests a potential role for TRPM4 in metastasis regulation through analogous mechanisms. Matrix metalloproteinases 1/2 (MMP1/2) have been identified as critical effector molecules in tumor invasion. MMP1 may contribute to the formation of an immunosuppressive microenvironment by remodeling the extracellular matrix (ECM) and regulating the TNFα/NF-κB pathway (Xu et al., 2025). MMP2 is transcriptionally suppressed by ZNF24 and regulated by the JNK/37LRP axis (Xing et al., 2024; Tian et al., 2025). It is hypothesized that TRPM4 is associated with the JNK pathway through calcium signaling, thereby upregulating MMP1/2 expression and enhancing the ECM degradation capacity of BLCA cells. In addition, the transforming growth factor-beta (TGF-β) pathway has been identified as a critical regulator of EMT and metastasis. RGS3 has been shown to promote TGF-β signaling by facilitating SMAD2/3 phosphorylation through ARID3B (Wang et al., 2025b). In addition, mechanical stress-induced cell deformation has been observed to induce TGF-β expression (Liu et al., 2026a). As an ion channel, TRPM4 may synergize with the TGF-β pathway to promote EMT by regulating membrane potential and cellular tension. At the metastatic structural level, TRPM4 may participate in the formation and stabilization of pseudopodia/invasive foci, in which integrin and Src kinase-mediated signaling play a crucial role (Hao et al., 2025). Concurrently, the secretion of extracellular vesicles by tumor cells has been demonstrated to remodel the distant microenvironment, thereby establishing a “pre-metastatic niche” (Li et al., 2025). TRPM4’s potential involvement in this process is suggested by its role in regulating vesicle release and content sorting. However, further experimental studies are needed to elucidate the exact mechanism by which TRPM4 influences BLCA.

We also founded that TRPM4-overexpressing epithelial cells interact with the tumor microenvironment through the MIF/MK signaling pathways. In the MIF pathway, epithelial-secreted MIF binds immune cell receptors, activating B-cell NF-κB to induce immunosuppressive cell infiltration, upregulate TRPM4 (via inflammatory feedback), and promote angiogenesis (Wang et al., 2022b). In the MK pathway, epithelial-secreted MK binds its receptor, activating FAK/Src and enhancing cell adhesion and matrix degradation. TRPM4 may influence these pathways by modulating membrane potential and ion gradients, but this hypothesis requires validation through functional experiments.

UNC93B1, an endosomal transmembrane protein critical for Toll-like receptor (TLR) 3/7/9 trafficking and activation, enhances TLR-mediated antigen presentation, thereby initiating antitumor immune responses and correlating with favorable prognoses in patients with BLCA (Ishida et al., 2021). UNC93B1’s regulatory role in innate immunity may be further modulated by TRPM4 through intracellular ion homeostasis, indirectly influencing TLR signaling pathways (Tohme et al., 2020). Conversely, FAM193B, previously implicated in renal carcinoma proliferation via the MAPK/ERK and PI3K/AKT pathways, exhibits a novel protective function in BLCA through EMT inhibition (Xie et al., 2021). The regulatory role of UNC93B1 in innate immunity may be influenced by intracellular ion homeostasis, and TRPM4, as an ion channel, may be involved in this process (Zhao et al., 2022); however, it remains unclear whether there is a direct functional link between the two. The potential synergistic interaction between FAM193B and TRPM4 also warrants further investigation. For example, it is necessary to determine whether TRPM4-mediated ion fluxes affect the activity of enzymes involved in lipid metabolism and whether FAM193B maintains metabolic balance through the formation of lipid droplets. The validation of these hypotheses necessitates rigorous testing through metabolomics and functional experiments. Mechanistically, FAM193B may synergize with TRPM4, thereby regulating lipid metabolism: TRPM4-mediated sodium signaling affects lipid synthesis enzyme activity, whereas FAM193B maintains lipid metabolic balance by modulating lipodroplet formation. Collectively, these findings reveal a coordinated network in which UNC93B1 and FAM193B suppress tumor malignancy through immune (via TLR activation) and lipid (via metabolic homeostasis) pathways, respectively. Both factors interact with TRPM4, thereby establishing a synergistic axis that restrains cancer progression. This dual regulatory mechanism emphasizes the significance of maintaining cellular homeostasis in tumor suppression and highlights potential therapeutic targets for intervention in BLCA.

POGLUT3 modulates bladder cancer (BLCA) progression through the regulation of the Notch signaling pathway. As an O-glucosyltransferase, POGLUT3 post-translationally modifies Notch receptors, thereby potentially influencing tumorigenic processes such as cell proliferation and differentiation (Pagliaro et al., 2020; Mehboob and Lang, 2021). RUNX2, a master transcription factor governing osteoblast differentiation, has recently been implicated in various solid tumors. Emerging evidence suggests that RUNX2 promotes BLCA invasion and metastasis by transcriptionally activating matrix metalloproteinases (MMPs) (Liu et al., 2020; Xiao et al., 2022; Lin, 2023). A potential synergistic interaction exists between these two factors: POGLUT3 may stabilize the RUNX2 protein via glycosylation, while RUNX2 may transcriptionally activate downstream effectors. This crosstalk may collectively drive the malignant phenotype of BLCA. Further experimental validation is required to elucidate the precise molecular mechanisms, which may offer novel therapeutic insights for BLCA treatment.

FBN1, a core component of the ECM, maintains its structural integrity and may influence the release of growth factors, such as TGF-β (Arous and Wehrle-Haller, 2017), with mutations linked to Marfan syndrome (Arnaud et al., 2021); in BLCA, elevated FBN1 expression may remodel the ECM microenvironment, where its degradation products activate TGF-β to induce EMT; altered ECM structure enhances integrin-mediated adhesion signaling, and FAK activation combined with TRPM4-mediated calcium influx may contribute to cell migration. TRPM4 further influences ECM cross-linking by modulating extracellular sodium ion concentrations. Conversely, MAP1B, a microtubule-associated protein, plays a key role in cell shape maintenance, organelle transport, and migration by stabilizing the microtubule cytoskeleton, with established importance in neurological disorders (Feng and Jiang, 2020; Tan et al., 2024). Based on the ion channel function of TRPM4 and the microtubule-binding characteristics of MAP1B, we postulate that these two proteins may constitute an “ion signaling–cytoskeleton” regulatory axis. Nevertheless, whether TRPM4 directly modulates the microtubule-binding activity of MAP1B, and whether this interaction plays a functional role in the metastasis of BLCA, require validation through *in vitro* functional assays, such as ion flux measurements and cell migration assays.

In the present study, we developed a prognostic risk scoring model for patients with BLCA according to prognostic gene expression. By integrating the expression levels of multiple prognostic genes, the model quantifies their cumulative aberrant effects into a single risk score, enabling clear stratification of patients into distinct high- and low-risk groups (Chen et al., 2022). Patients in the HRG had a significantly lower overall survival rate than those in the LRG, suggesting the model’s strong prognostic predictive capability. This model provides clinicians with a convenient prognostic assessment tool, enabling more precise identification of high-risk patients with poor prognosis. This development enables the formulation of more assertive treatment plans and close follow-up strategies for these patients. Concurrently, for low-risk patients, the model effectively circumvents the physical and psychological stresses associated with overtreatment, thereby enhancing their quality of life and optimizing individualized treatment decisions.

In the HRG, we observed significant enrichment in the ARVC pathway. Emerging evidence indicates that aberrant desmoplakin expression in BLCA compromises urothelial integrity, thereby promoting tumor cell migration (Vite et al., 2020). The enrichment of this pathway in our study may reflect ARVC-like cell junction defects in BLCA cells, promoting tumor spread by reducing intercellular adhesion. Further, aberrant calcium signaling within this pathway can elicit subsequent activation of pro-cancerous pathways. In the LRG, significant enrichment was observed in the drug metabolism–cytochrome P450 pathway. Cytochrome P450 enzymes are critical for drug metabolism, and their expression levels in BLCA exhibit a strong correlation with chemotherapy sensitivity (Mao et al., 2024). In this study, the drug metabolism–cytochrome P450 pathway was enriched in the LRG, suggesting that it may reduce drug resistance by efficiently metabolizing chemotherapy drugs. This finding is consistent with the typically favorable prognosis of low-risk patients, providing a molecular basis for selecting chemotherapy regimens in clinical practice. A notable observation was the substantial enrichment of the melanoma pathway in the HRG. Although melanoma originates from melanocytes and BLCA from urothelial cells, these two malignancies share multiple molecular hallmarks of invasive behavior, including sustained activation of the MAPK/ERK and PI3K-Akt signaling pathways, enhanced matrix metalloproteinase activity, and increased invasive potential (Wellbrock and Arozarena, 2016; Kudelski et al., 2023; Rezaei et al., 2023). In the context of BLCA, these processes are closely linked to TRPM4-mediated calcium influx and subsequent downstream transcriptional reprogramming. Notably, RUNX2, a prognostic gene identified in this study, has been reported to promote melanoma metastasis by regulating MMP expression and EMT (Dalle Carbonare et al., 2024). This parallel phenomenon suggests that the TRPM4/RUNX2 axis may activate melanoma-associated transcriptional programs in BLCA, thereby contributing to a poor prognosis. Consequently, the enrichment of melanoma pathways does not signify a histological transformation; rather, it reflects convergent functional phenotypes across diverse cancer types.

Subsequent drug-sensitivity analyses identified 112 compounds with significantly different IC50 values between the HRG and LRG (*p* < 0.05), thereby highlighting marked variations in drug response among patients in different risk groups. Notably, the negative correlation between POGLUT3 and TGX221 suggests that increased POGLUT3 expression may be associated with greater cellular resistance to TGX221 (Cen et al., 2025). This could occur because elevated POGLUT3 expression triggers specific intracellular defense mechanisms, enabling cells to resist the inhibitory effects of TGX221. However, the expression levels of the POGLUT3 gene have been shown to correlate positively with those of WZ3105, suggesting that their expression levels tend to change synchronously. This finding suggests that increased POGLUT3 expression may enhance the efficacy of WZ3105. This phenomenon may be due to elevated POGLUT3 expression, which creates a more favorable cellular environment for WZ3105s action, thereby potentiating its inhibitory effect on cancer cells. These findings suggest that POGLUT3 may regulate BLCA cells’ sensitivity to specific drugs, underscoring the need to consider individual gene expression variations during treatment.

The IHC analysis validated the expression of prognostic genes at the protein level in BLCA tissues. The prognostic gene UNC93B1 exhibited strong positive expression in BLCA tissues, and was primarily localized to the cell membrane and cytoplasm, with a positive cell proportion exceeding. This finding is consistent with the transcriptomic analysis of UNC93B1. Further, FAM193B showed intense nuclear expression in BLCA tissues, with over 75% of cells being positive. This nuclear expression pattern may indicate a role for FAM193B in cell cycle regulation and gene expression. These results validate the high expression of UNC93B1 and FAM193B in BLCA and provide crucial evidence for subsequent functional validation experiments.

These findings, along with the qRT–PCR results for key genes in BLCA tissues, collectively support the hypothesis that the aforementioned genes are critically involved in BLCA pathogenesis and progression. These findings provide stronger support for subsequent in-depth studies on the functional mechanisms of prognostic genes and the development of diagnostic biomarkers or therapeutic targets.

Nevertheless, this study has some limitations. First, the specific molecular mechanisms underlying these prognostic genes in BLCA remain poorly elucidated, and all hypothetical molecular mechanisms (including interactions between TRPM4 and JNK, TGF-β, POGLUT3, RUNX2, etc.) require subsequent functional validation through experimental studies. Second, although the risk model demonstrated strong prognostic predictive ability, its clinical utility and efficacy for targeted drugs require further validation. Thus, future research should focus on investigating the functions and therapeutic applications of these prognostic genes to further confirm their clinical value.

This study systematically analyzed TRPM4’s expression, biological functions, and regulatory mechanisms in BLCA by integrating single-cell and transcriptomic data. High TRPM4 expression in BLCA is closely associated with tumor progression and prognosis, potentially affecting tumor cell biology through multiple mechanisms, including regulation of intracellular sodium homeostasis, intercellular communication, and the immune microenvironment. In addition, the risk-scoring model, GSEA analysis, and drug-sensitivity study provide key references for personalized treatment in BLCA. Future research should further explore TRPM4’s specific mechanisms and develop TRPM4-targeted therapies to improve treatment efficacy and survival rates in patients with BLCA.

## Data Availability

The datasets presented in this study can be found in online repositories. The names of the repository/repositories and accession number(s) can be found in the article/Supplementary Material.

## Supplementary material

SUPPLEMENTARY FIGURE 1 (A,B) Quality control metrics comparison of single - cell RNA - seq data between Normal and Tumor tissues. **(C)** The scatter plot depicts the relationship between the average expression level of genes and the standardized variance. The black dots represent non-variable genes, and the orange dots represent variable genes. **(D,E)** PCA scatter plot of samples based on gene expression profiles. The plot illustrates the distribution of normal (purple) and tumor (pink) samples along the first two principal components (PC1 and PC2). **(F)** Differential gene expression analysis in epidermal cells expressing TRPM4.

SUPPLEMENTARY FIGURE 2 The specific interaction bubble chart of signaling pathway ligand-receptor pairs across different cell types suggests that three MIF-related ligand-receptors are closely associated with epithelial cells **(A)**, while MK-related pathways are more prevalent **(B)**.

SUPPLEMENTARY FIGURE 3
**(A)** The AUC curve indicates that the risk prediction model has good predictive performance. **(B-D)** The risk model demonstrates consistent predictive performance when evaluated through the validation set. **(E,F)** The KM survival analysis curves and AUC curves obtained from the validation set indicate consistent results with the training set.

SUPPLEMENTARY FIGURE 4 The **(A)** 1-, **(B)** 3-, and **(C)** 5-year time-dependent ROC curves comparing the predictive performance of clinical-pathological indicators, genetic features, and a combined model of both (nomogram).

SUPPLEMENTARY TABLE 1 Baseline characteristics of patient samples and qRT-PCR primer information.

SUPPLEMENTARY TABLE 2 TRPM4-related DEGs data.

SUPPLEMENTARY TABLE 3 DEGs data in epithelial cells.

SUPPLEMENTARY TABLE 4 Core cell gene names.

SUPPLEMENTARY TABLE 5 Candidate genes names.

SUPPLEMENTARY TABLE 6 Univariate regression analysis data.

SUPPLEMENTARY TABLE 7 matrix-related pathways data.

SUPPLEMENTARY TABLE 8 Drug sensitivity analysis results based on R language.

## Glossary

BLCA: Bladder cancer
TRPM4: Transient receptor potential cation channel subfamily M, member 4
UNC93B1: Protein unc-93 homolog B1
FAM193B: Family with sequence similarity 193 member B
POGLUT3: Protein O-glucosyltransferase 3
FBN1: Fibrillin-1
MAP1B: Microtubule-associated protein 1B
RUNX2: RUNX family transcription factor 2
DEGs: Differentially expressed genes
LEADR: Long epithelial alu-interacting differentiation-related
ITGA8: Integrin subunit alpha 8
MEN1: Multiple endocrine neoplasia type 1
ERO1A: Endoplasmic reticulum oxidoreductase 1 alpha
ALOX5: Arachidonate 5-lipoxygenase
hdWGCNA: High-dimensional weighted gene co-expression network analysis
NAT: Normal adjacent tissue
PCA: Principal component analysis
PCs: Principal components
UMAP: Uniform manifold approximation and projection
kME: Key module eigengene-based connectivity
SCENIC: Single-Cell Regulatory Network Inference and Clustering
TFs: Transcription factors
AUC: Area under the curve
PH: Proportional hazards
HRG: High-risk group
LRG: Low-risk group
GSEA: Gene set enrichment analysis
IHC: Immunohistochemistry
HPA: Human Protein Atlas
qRT-PCR: Quantitative real-time polymerase chain reaction
NK: Natural killer cells
MIF: Macrophage migration inhibitory factor
MK, MDK: Midkine
ARVC: Arrhythmogenic right ventricular cardiomyopathy
EMT: Epithelial-mesenchymal transition
MMP: Matrix metalloproteinases
ECM: Extracellular matrix
TLR: Toll-like receptor
