# Supplementary material for: Integrate single-cell and transcriptome analyses to explore the prognostic genes related to TRPM4 in bladder cancer
Source: Front Bioeng Biotechnol. 2026 Apr 13;14:1773551. doi: 10.3389/fbioe.2026.1773551 (PMC13111456; doi:10.3389/fbioe.2026.1773551)
Supplement: Supplementary file 3 [file Table1.docx]

**Baseline characteristics**

| Variables | Number of patients(n=10) | Proportion(%) |
| --- | --- | --- |
| Gender | | |
| Male | 9 | 90 |
| Female | 1 | 10 |
| Age(Years) | |  |
| ＜60 | 2 | 20 |
| ≥60 | 8 | 80 |
| Tumor Size(mm) | |  |
| ＜20 | 2 | 20 |
| ≥20 | 8 | 80 |
| T Stage | |  |
| Ta-T1 | 8 | 80 |
| T2-T4 | 2 | 20 |
| N Stage | |  |
| N0-N1 | 9 | 90 |
| N2-N3 | 1 | 10 |
| Grade | |  |
| High | 6 | 60 |
| Low | 4 | 40 |
| HER2 expression（IHC score） |  |  |
| ＜2+ | 6 | 60 |
| ≥2+ | 4 | 40 |

**Primer sequences**

| Gene | Forward primer | Reverse primer |
| --- | --- | --- |
| UNC93B1 | TGCTCACCTACGGCGTCTA | GATGGGAGTCACGTTGATGC |
| FAM193B | AGTGCAGAAACCCCACCAAA | ATGACGAACCCAACCTGGTG |
| POGLUT3 | TGGAGCATCGAAAAGTCAATGG | CATACGTTGGAAGGACAACATCT |
| FBN1 | TTTCTGCTCACTGGATGTGC | ACAGCAGAAGCTGGGAAGAA |
| MAP1B | AATTCCTGGGCAAACTGGTCT | AGAGCCGGACTGGAGAATGA |
| RUNX2 | AGTGCGGTGCAAACTTTCTC | TGACTCTGTTGGTCTCGGTG |
| GAPDH | GGAGCGAGATCCCTCCAAAAT | GGCTGTTGTCATACTTCTCATGG |
