# Supplementary figures and images for: Integrate single-cell and transcriptome analyses to explore the prognostic genes related to TRPM4 in bladder cancer
Source: Front Bioeng Biotechnol. 2026 Apr 13;14:1773551. doi: 10.3389/fbioe.2026.1773551 (PMC13111456; doi:10.3389/fbioe.2026.1773551)

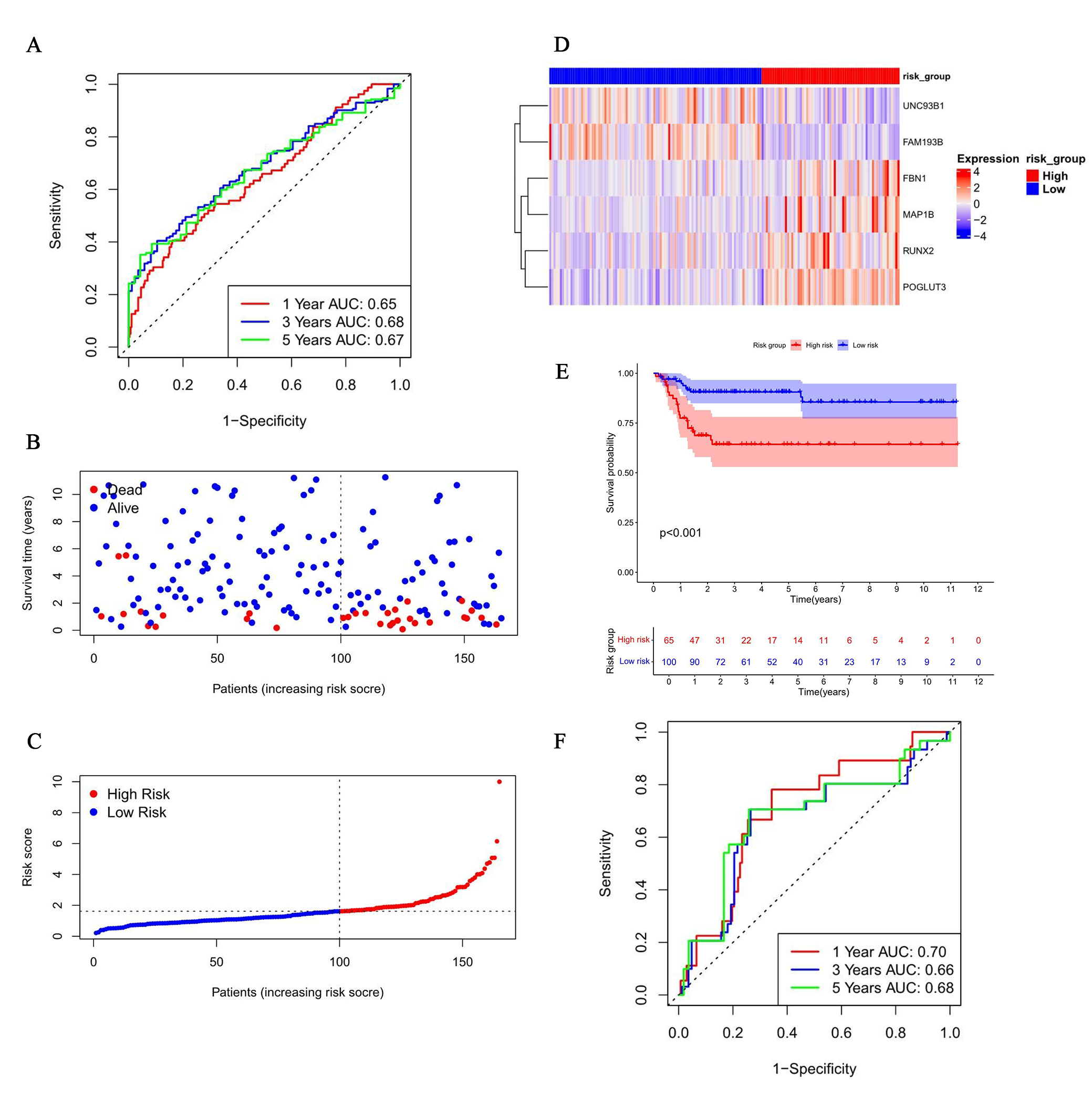

Supplement: Supplementary file 4 [file Image3.tif]

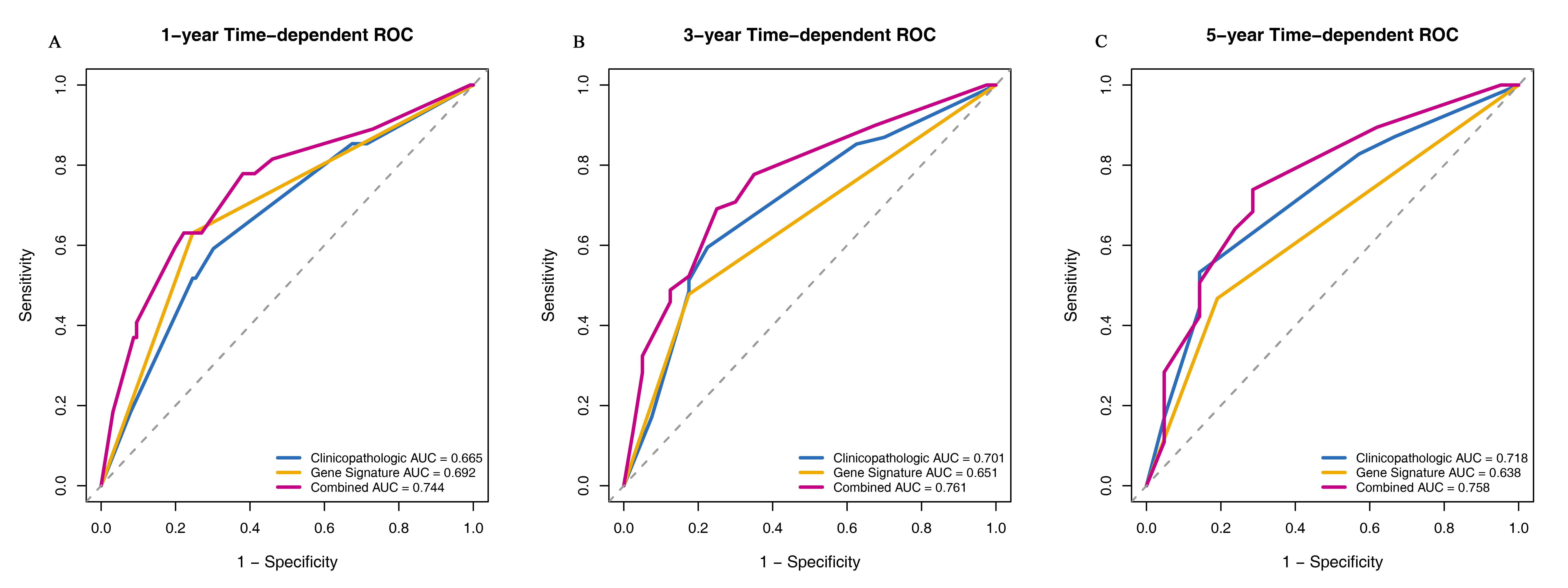

Supplement: Supplementary file 5 [file Image4.tif]

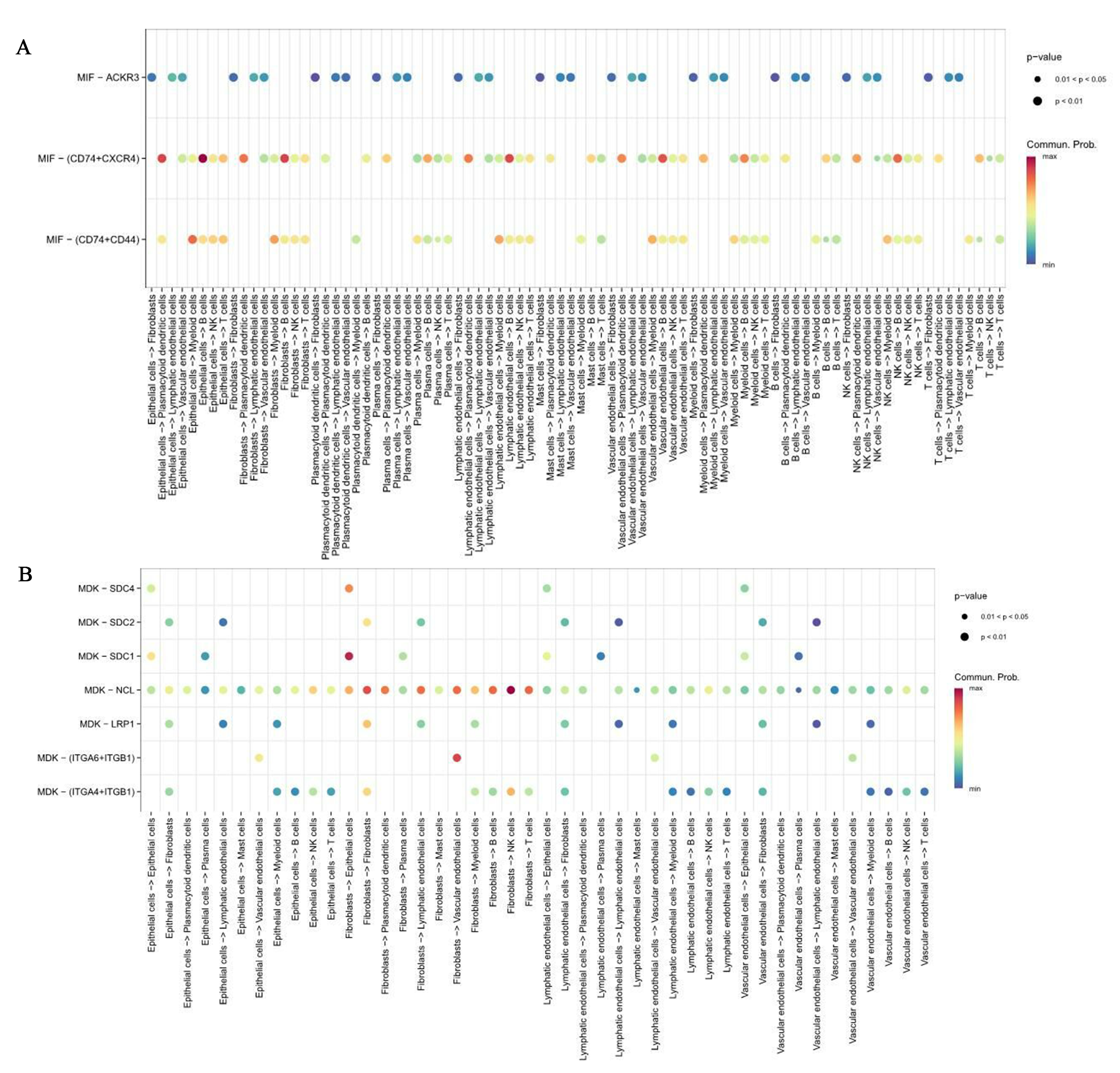

Supplement: Supplementary file 6 [file Image2.tif]

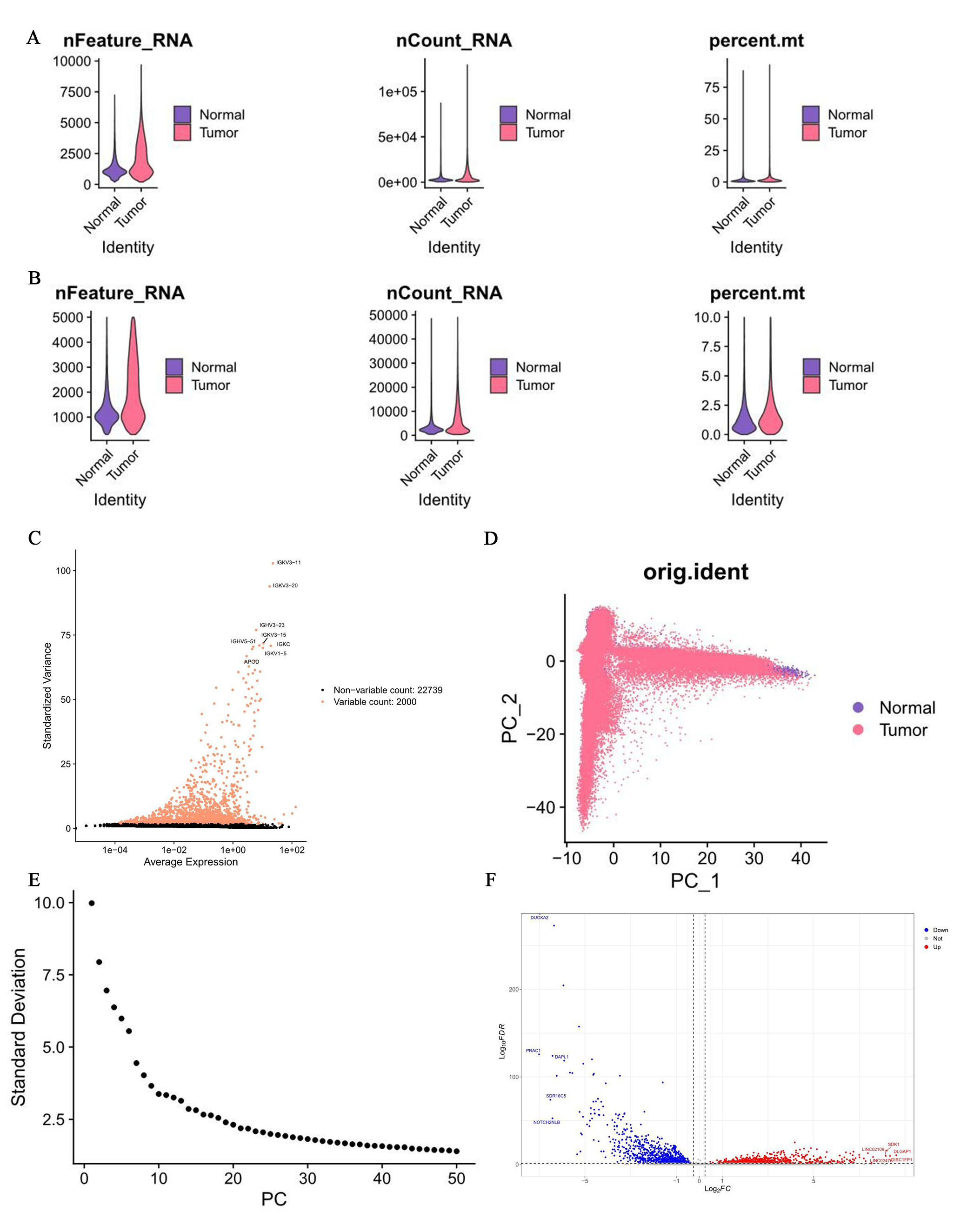

Supplement: Supplementary file 7 [file Image1.tif]
